# Early Cessation of Renal Replacement Therapy in Theophylline Toxicity: A Case Report With Literature Review

**DOI:** 10.7759/cureus.88081

**Published:** 2025-07-16

**Authors:** Shungo Kakiuchi, Haruki Mizuno, Kanako Irei, Minoru Hayashi

**Affiliations:** 1 Department of Emergency Medicine, Fukui Prefectural Hospital, Fukui, JPN

**Keywords:** case report, cessation, drug poisoning, renal replacement therapy, theophylline toxicity

## Abstract

Theophylline toxicity often results in severe neurological and cardiovascular complications and may require renal replacement therapy (RRT) in life-threatening situations. However, guidance on when it is safe to discontinue RRT remains limited.

We report the case of an 85-year-old woman who developed status epilepticus following theophylline overdose, with a peak serum level of 119.8 mg/L. She was initially hemodynamically unstable and received continuous hemodiafiltration (CHDF). After clinical stabilization and a reduction in serum theophylline to 24.7 mg/L, CHDF was discontinued despite mild tachycardia and low-dose catecholamine use. The patient remained stable without rebound toxicity and recovered uneventfully. This case highlights that early discontinuation of RRT may be feasible even when theophylline levels exceed traditional thresholds, provided there is sustained clinical improvement, including stability despite mild tachycardia and low-dose catecholamine support.

## Introduction

Theophylline has been widely used as a bronchodilator for the treatment of asthma and chronic obstructive pulmonary disease (COPD) [1]. However, due to its narrow therapeutic index, theophylline toxicity remains a significant clinical concern. Overdose can lead to severe neurological, cardiovascular, and metabolic complications [2,3]. Although the incidence of theophylline poisoning has substantially declined since the 1980s, only 128 exposures and one fatality were reported to United States poison centers in 2022 [4].

Nonetheless, severe and life-threatening cases continue to be observed in clinical practice, particularly among older adults. Renal replacement therapy (RRT), such as hemodialysis or hemofiltration, is considered necessary when the serum theophylline concentration exceeds 100 mg/L (888 μmol/L), seizures occur, or the patient develops shock, to enhance drug elimination [5].

The Extracorporeal Treatments in Poisoning (EXTRIP) workgroup guidelines recommend the discontinuation of extracorporeal therapies when clinical improvement is observed or when the serum theophylline level falls below 15 mg/L (83 μmol/L). However, the guidelines do not provide specific criteria for what constitutes clinical improvement such as thresholds for vital signs. Moreover, the rationale section does not cite any references related to vital signs. With reference to serum theophylline concentrations, the recommendations are primarily based on case series from the 1970s and 1980s, which the guideline grading system classifies as having a "very low" level of evidence [6].

Given the substantial evolution of intensive care practices and recent advancements in both critical care and renal replacement therapy, it is important to reassess whether these traditional thresholds remain appropriate in modern clinical settings.

Here, we report a case of acute theophylline toxicity in which RRT was discontinued at a serum theophylline concentration of 24.7 mg/L (137.1 μmol/L), despite the presence of persistent tachycardia around 100 bpm and ongoing low-dose catecholamine support. This case suggests that, in select situations, RRT may be safely discontinued above the currently recommended threshold. The CARE (CAse REport) guidelines were used for this case report.

## Case presentation

An 85-year-old woman with a history of bronchial asthma, dyslipidemia, and hypertension was found convulsing at home. She had been prescribed sustained-release theophylline, and her family reported finding empty medication blister packs, containing a total of 20 doses, each with 100 mg, in her room, suggesting a significant overdose.

**Figure 1:**
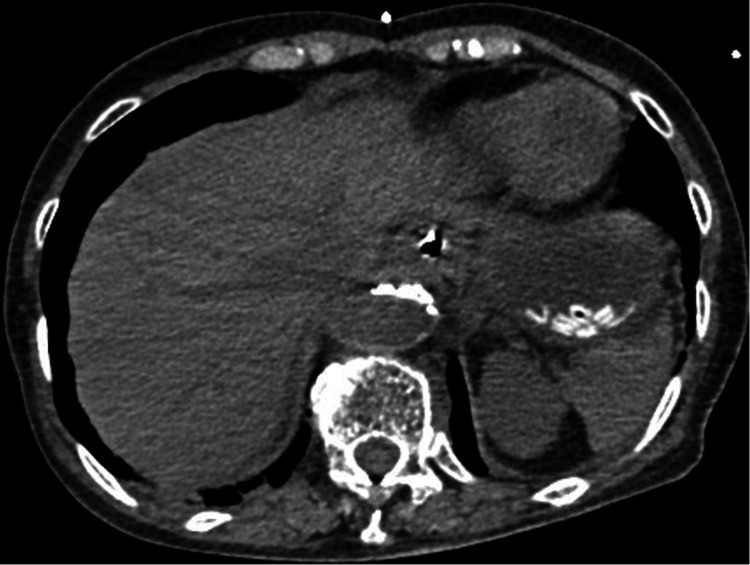
Abdominal CT scan showing multiple high-density materials in the stomach, consistent with ingested tablets.

Upon arrival at the emergency department (ED), she was in status epilepticus with a heart rate of 142 beats per minute (bpm), indicating sinus tachycardia, blood pressure of 77/36 mmHg, respiratory rate of 30 breaths per minute, body temperature of 36.5 °C, and an oxygen saturation of 97% on room air. An initial electrocardiogram (ECG) showed narrow-complex sinus tachycardia at 170 bpm without QT prolongation. Venous blood gas analysis revealed a pH of 6.74, pCO₂ of 112 mmHg, HCO₃⁻ of 16.7 mmol/L, lactate of 17.6 mmol/L, glucose of 9.5 mmol/L, and serum potassium of 6.6 mEq/L, indicating severe metabolic and respiratory acidosis. Abdominal computed tomography (CT) was performed to investigate the cause of severe metabolic and respiratory acidosis and revealed multiple high-density spots in the stomach, consistent with ingested pills (Figure 1). Given the serum theophylline concentration of 119.8 mg/L (665.3 μmol/L), a diagnosis of acute theophylline toxicity was made.

**Figure 2:**
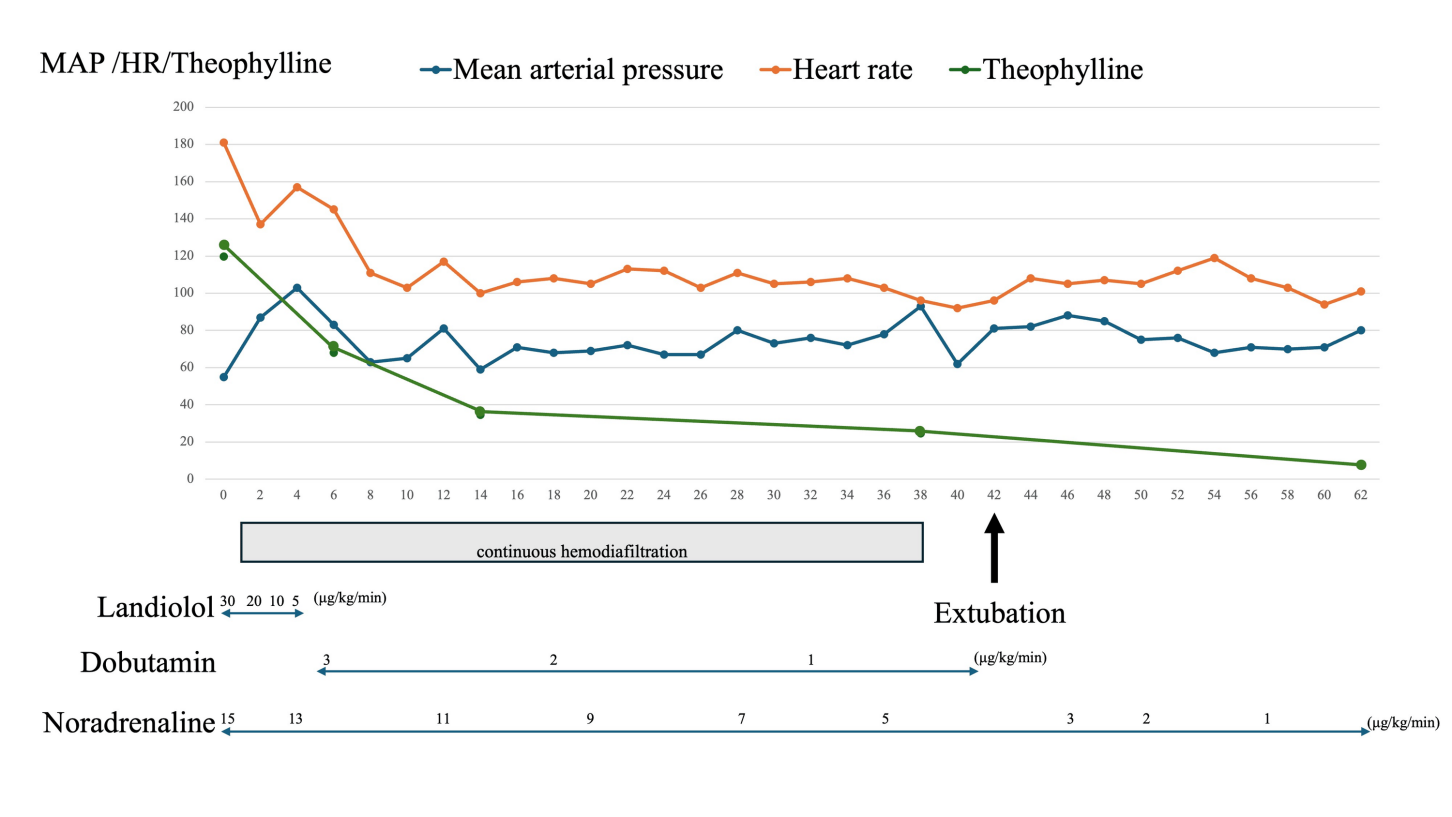
Clinical course in the ICU, including serum theophylline concentrations, heart rate, and major interventions Continuous hemodiafiltration was initiated 1 hour after ICU admission and discontinued at 38 hours, when the serum theophylline concentration was 24.7 mg/L. No rebound was observed following discontinuation, and the patient remained hemodynamically stable. MAP: mean arterial pressure(mmHg); HR: heart rate(bpm); Theophylline: the serum theophylline concentration (mg/L)

Emergency treatment was initiated with intravenous diazepam (5 mg, administered twice), but the seizures did not resolve. Subsequently, midazolam (5 mg) was administered, which successfully terminated the seizures. To secure the airway in the context of status epilepticus management, endotracheal intubation was performed. To manage hemodynamic instability, norepinephrine (10 mcg/min) was started. The patient was admitted to the intensive care unit (ICU), where gastric lavage and 50 g of activated charcoal were administered. Given the severity of the intoxication, Continuous hemodiafiltration (CHDF) was initiated approximately one hour after ICU admission. CHDF parameters included a blood flow rate of 100 mL/min, dialysate flow rate of 800 mL/h, replacement fluid at 400 mL/h, and heparin sodium for anticoagulation. The clinical course following ICU admission is summarized in Figure 2, which illustrates changes in vital signs (heart rate and blood pressure), serum theophylline concentrations, and therapeutic interventions.

Six hours after ICU admission, CHDF reduced the theophylline concentration to 68.0 mg/L (377.9 μmol/L), and the heart rate improved. Fourteen hours after ICU admission, the theophylline concentration further declined to 34.7 mg/L (192.8 μmol/L). By 38 hours, it had decreased to 24.7 mg/L (137.3 μmol/L). Although the patient had persistent tachycardia around 100 bpm and required low-dose catecholamine support, she remained clinically stable with a mean arterial pressure (MAP) of at least 65 mmHg and no subjective symptoms. Based on these factors, CHDF was discontinued despite the theophylline concentration exceeding the guideline-recommended threshold of 15 mg/L (83 μmol/L). Following the cessation of CHDF, serum theophylline levels continued to decline spontaneously, reaching 7.7 mg/L (42.8 μmol/L) at 62 hours post-admission.

Landiolol was initially administered to control theophylline-induced tachycardia, but it was discontinued early in the clinical course. As the patient’s condition stabilized, catecholamine support was weaned and completely discontinued approximately 62 hours after ICU admission.

Although the patient initially presented in status epilepticus, seizures were successfully terminated with bolus administration of benzodiazepines, and no recurrence was observed. Continuous infusion of midazolam or propofol was not required for seizure control, and antiepileptic drugs were not administered during the ICU stay.

The patient was successfully extubated 42 hours after ICU admission and continued to show stable clinical improvement. She remained under observation in the ICU for monitoring and supportive care and was transferred to a general ward on day 7.

## Discussion

In this case, RRT was discontinued at a theophylline concentration of 24.7 mg/L (137.3 μmol/L), which exceeded the commonly recommended threshold of 15 mg/L(83 μmol/L). Nevertheless, there was no clinical deterioration or rebound increase in serum theophylline levels following the cessation of RRT. This case suggests that RRT might be safely discontinued even at serum theophylline concentrations higher than the traditionally recommended threshold. These objective findings may serve as a useful reference for clinicians considering RRT discontinuation, potentially contributing to the development of more concrete clinical criteria in the future. With advances in critical care, it is necessary to reassess whether these historical thresholds remain appropriate.

We searched PubMed and Google Scholar for case reports of theophylline toxicity treated with RRT published between 2015 and 2025, following the release of the EXTRIP guidelines. The search was limited to adult patients (aged 18 years or older) and yielded a total of six cases (Table 1) [7-12]. Four of the six case reports did not include specific data regarding the patient's vital signs [7,8,10,11]. Only two case reports have documented vital signs at the time of RRT discontinuation, with heart rates in the 80s and blood pressure recorded at 72/67 mmHg [9,12]. In one case report, the theophylline concentration at the time of RRT discontinuation was documented as 10.5 mg/L(58.3μmol/L) [11]. Three studies documented the lowest recorded theophylline concentrations, which ranged from 5.9 to 10.5 mg/L (32.7 to 58.3μmol/L) [9,10,12]. However, it remains unclear whether these lowest concentrations corresponded to the actual time of RRT discontinuation. The absence of this information makes it unclear whether current clinical practice allows for RRT discontinuation at a theophylline concentration above the guideline-recommended threshold of 15 mg/L (83 μmol/L). In contrast, our case documents the patient’s clinical status-including persistent tachycardia around 100 bpm under low-dose catecholamine support, at the time of CHDF discontinuation when the serum theophylline level was still 24.7 mg/L (137.3 μmol/L). We report a case in which RRT was stopped at a theophylline level above the recommended threshold, with specific vital signs recorded at the time-something not shown in previous reports.

**Table 1:** Summary of reported cases of theophylline toxicity treated with renal replacement therapy (RRT) NA: not available; RRT: renal replacement therapy; HD: hemodialysis; CHD: continuous hemodialysis; CHDF: continuous hemodiafiltration; CRRT: continuous renal replacement therapy; MAP: mean arterial pressure (mmHg); BP: blood pressure (mmHg); HR: heart rate (bpm); †Our case refers to the patient we describe in this report. ‡“Acute on chronic” indicates a case in which the patient was on chronic theophylline therapy and subsequently ingested an excessive dose. §“Lowest Recorded Level” refers to the lowest serum theophylline concentration documented in the case report; however, it was unclear whether ECTR was discontinued at that level.

Case	Age	Sex	Presenting Symptom	Type of Overdose	Initial Theophylline Level (mg/L)	Level at RRT Discontinuation (mg/L)‡	Lowest Recorded Level (mg/L)§	Vital Signs at Discontinuation	RRT Modality
Our case†	85	Female	Seizure	Acute on Chronic‡	119.8	24.7	7.7	HR110, MAP65	CHDF
Kong et al. [8]	83	Male	Seizure	Acute on Chronic	80.7	NA	NA	NA	HD
Joyce et al. [9]	30	Female	Seizure	Acute on Chronic	>40	NA	NA	NA	CHD
Ichikawa et al. [10]	76	Male	Shock	Acute on Chronic	38.5	NA	8.2	HR80	CHDF
Jinah et al. [11]	77	Female	Shock	Chronic	31.7	NA	5.9	NA	CHDF
Kommineni et al. [12]	69	Female	Seizure	Acute on Chronic	59.7	NA	NA	NA	HD
Sim et al. [5]	73	Male	Shock	Chronic	>40	10.5	10.5	BP72/67, HR87	CRRT

It may be appropriate to reconsider whether the current recommended threshold of 15 mg/L (83 μmol/L) for discontinuing RRT is universally necessary. However, this is a single case report, and its findings may not be generalizable to other patients or clinical settings. Further studies with larger sample sizes are needed to confirm our observations.

## Conclusions

Theophylline toxicity can be life-threatening, often requiring RRT for rapid elimination. While guidelines recommend discontinuing RRT once clinical improvement is apparent or theophylline levels fall below 15 mg/L, these recommendations are based on limited data. In this case, RRT was discontinued at 24.7 mg/L despite the presence of tachycardia and the need for low-dose catecholamine support, with no rebound toxicity observed. This suggests that the discontinuation of RRT may be safe at higher theophylline levels.
